# Supplementary material for: Experiences of women living with Polycystic Ovary Syndrome: A pilot case-control, single-cycle, daily Menstrual Cycle Diary study during the SARS-CoV-2 pandemic
Source: PLoS One. 2023 Dec 29;18(12):e0296377. doi: 10.1371/journal.pone.0296377 (PMC10756515; doi:10.1371/journal.pone.0296377)
Supplement: Attachment — Submitted filename: Peer Review.PLoS ONE.PCOS during Covid.docx [file pone.0296377.s001.docx]

This research article sought to examine the menstrual cycle and daily life experiences of women with PCOS as compared to women without PCOS during the Covid-19 pandemic. PCOS is an important and needed area of research. However, this article presents several flaws.

First, requiring month-apart menstrual cycles severely limited your PCOS population, especially those with most disturbing PCOS symptoms. This study most likely sampled the milder phenotype (a much smaller prevalence), and oligo- ovulatory women with PCOS exhibit milder symptoms. There is also research showing that depressive levels among this phenotype are comparable to women without PCOS due to lack of or less obesity, hirsutism, alopecia, etc. **This was not a true comparison of women with PCOS in its most prevalent form and women without PCOS.**

As such, the conclusions are misrepresented and overstated. Most women with PCOS (phenotypes A and B) do not have similar cycle lengths and ovulatory characteristics. This needs to be stated in the intro and conclusion given the chosen group of women with PCOS. Anovulatory women with PCOS are more often amenorrheic and present with longer cycle duration.

The authors state this is a pilot study, but there are no measures of feasibility and acceptability. Although this study included a control group, it is more of a cross-sectional design due to its results of prevalence, not predictors of outcome measures.

Self-worth, as a concept, was not properly defined. As written, the study measured self-image. Outside stresses as defined by the MOS2 was not specified. There is only a signal of significant difference on outside stressors. Without this definition, the discussion stretches beyond the results. Also, there is a notable difference between statistical and clinical significance. This study has not indication or description of clinical significance, especially among those who represent about 4% of women with PCOS. Thus, this study severely lacks external validity.

Lines 363: Information introduced in the discussion that should be placed in the background information. Also, the purpose of the study was not a comparison of stressful situations (i.e., number or type) and stressful situations were not defined (as stated above). Also, it is discussed that coping mechanisms mitigate the relationship between stress and mental disorders. Coping mechanisms were not measured. Types of stress were not measured. There was no difference detected with symptoms of anxiety and depression.

Lines 469-475 of the conclusion are irrelevant to the study. The women with PCOS were similar because women with PCOS similar to those without PCOS were purposefully studied. Feasibility was not supported because it was not measured.
